# Selection of HIV Envelope Strains for Standardized Assessments of Vaccine-Elicited Antibody-Dependent Cellular Cytotoxicity-Mediating Antibodies

**DOI:** 10.1128/JVI.01643-21

**Published:** 2022-01-26

**Authors:** Dieter Mielke, Sherry Stanfield-Oakley, Bhavesh Borate, Leigh H. Fisher, Katelyn Faircloth, Marina Tuyishime, Kelli Greene, Hongmei Gao, Carolyn Williamson, Lynn Morris, Christina Ochsenbauer, Georgia Tomaras, Barton F. Haynes, David Montefiori, Justin Pollara, Allan C. deCamp, Guido Ferrari

**Affiliations:** a Department of Surgery, Duke University grid.471396.egrid.26009.3d , Durham, North Carolina, USA; b Statistical Center for HIV/AIDS Research and Prevention, Fred Hutchinson Cancer Research Center, Seattle, Washington, USA; c Vaccine and Infectious Diseases Division, Fred Hutchinson Cancer Research Center, Seattle, Washington, USA; d Division of Medical Virology, Department of Pathology, University of Cape Town, Cape Town, South Africa; e National Health Laboratory Services, Johannesburg, South Africa; f Centre for the AIDS Programme of Research in South Africa (CAPRISA), University of KwaZulu Natal, Durban, South Africa; g HIV Virology Section, Center for HIV and STIs, National Institute for Communicable Diseases, Johannesburg, South Africa; h MRC Antibody Immunity Research Unit, University of Witwatersrand, Johannesburg, South Africa; i Department of Medicine, University of Alabama at Birmingham, Birmingham, Alabama, USA; j Department of Molecular Genetics and Microbiology, Duke University grid.471396.egrid.26009.3d , Durham, North Carolina, USA; k Department of Immunology, Duke University grid.471396.egrid.26009.3d , Durham, North Carolina, USA; l Duke Human Vaccine Institute, Duke University School of Medicine, Durham, North Carolina, USA; m Department of Medicine, Duke University grid.471396.egrid.26009.3d , Durham, North Carolina, USA; Icahn School of Medicine at Mount Sinai

**Keywords:** antibody dependent cellular cytotoxicity, human immunodeficiency virus

## Abstract

Antibody-dependent cellular cytotoxicity (ADCC) has been correlated with reduced risk of human immunodeficiency virus type 1 (HIV-1) infection in several preclinical vaccine trials and in the RV144 clinical trial, indicating that this is a relevant antibody function to study. Given the diversity of HIV-1, the breadth of vaccine-induced antibody responses is a critical parameter to understand if a universal vaccine is to be realized. Moreover, the breadth of ADCC responses can be influenced by different vaccine strategies and regimens, including adjuvants. Therefore, to accurately evaluate ADCC and to compare vaccine regimens, it is important to understand the range of HIV Envelope (Env) susceptibility to these responses. These evaluations have been limited because of the complexity of the assay and the lack of a comprehensive panel of viruses for the assessment of these humoral responses. Here, we used 29 HIV-1 infectious molecular clones (IMCs) representing different Envelope subtypes and circulating recombinant forms to characterize susceptibility to ADCC from antibodies in plasma from infected individuals, including 13 viremic individuals, 10 controllers, and six with broadly neutralizing antibody responses. We found in our panel that ADCC susceptibility of the IMCs in our panel did not cluster by subtype, infectivity, level of CD4 downregulation, level of shedding, or neutralization sensitivity. Using partitioning around medoids (PAM) clustering to distinguish smaller groups of IMCs with similar ADCC susceptibility, we identified nested panels of four to eight IMCs that broadly represent the ADCC susceptibility of the entire 29-IMC panel. These panels, together with reagents developed to specifically accommodate circulating viruses at the geographical sites of vaccine trials, will provide a powerful tool to harmonize ADCC data generated across different studies and to detect common themes of ADCC responses elicited by various vaccines.

**IMPORTANCE** Antibody-dependent cellular cytotoxicity (ADCC) responses were found to correlate with reduced risk of infection in the RV144 trial of the only human HIV-1 vaccine to show any efficacy to date. However, reagents to understand the breadth and magnitude of these responses across preclinical and clinical vaccine trials remain underdeveloped. In this study, we characterize HIV-1 infectious molecular clones encoding 29 distinct Envelope strains (Env-IMCs) to understand factors that impact virus susceptibility to ADCC and use statistical methods to identify smaller nested panels of four to eight Env-IMCs that accurately represent the full set. These reagents can be used as standardized reagents across studies to fully understand how ADCC may affect efficacy of future vaccine studies and how studies differ in the breadth of responses developed.

## INTRODUCTION

Antibody-dependent cellular cytotoxicity (ADCC) responses have been associated with lower viremia in human immunodeficiency virus type 1 (HIV-1)-infected individuals (1, 2), observed to be enriched in HIV-1 controllers (3, 4), and associated with curbing early simian immunodeficiency virus (SIV) viral replication in nonhuman primates (NHPs) (5–7). In addition, ADCC-mediating antibodies in breast milk have been correlated with reduced vertical transmission from viremic mothers (8). Consequently, there is substantial evidence to suggest that ADCC plays an important role in HIV-1 infection.

This is further supported by the results of the RV144 clinical trial, the only HIV-1 vaccine trial to date to show modest efficacy, which identified ADCC responses as a correlate of reduced risk of infection (9). Several preclinical trials support these observations. A pentavalent vaccine designed to improve antibody responses and enhance the protection observed in the RV144 trial and tested against a simian-human immunodeficiency virus (SHIV) challenge in NHPs again identified ADCC responses as a correlate of protection (10), and recently, ADCC responses elicited by a 6-valent vaccine were associated with decreased SHIV transmission risk (11). Conversely, the recent finding of lack of efficacy in the HVTN702 clinical trial brings into question the breadth of coverage that this vaccine could provide against circulating endemic HIV-1. As a result, ADCC responses elicited in future HIV-1 vaccine trials need to be methodically investigated.

Similarly to neutralization, assessments of HIV-1 vaccine-elicited ADCC-mediating antibody responses need to adequately address viral genetic and phenotypic diversity. Several large panels of pseudoviruses inclusive of numerous subtypes have been constructed and neutralization profiles comprehensively characterized using plasma from infected individuals and broadly neutralizing monoclonal antibodies (12–14). These studies revealed that variants have a range of neutralization susceptibility, referred to as tiers, with viruses clustering into one of four subgroups, as follows: very high (tier 1a), above average (tier 1b), moderate (tier 2), or low sensitivity (tier 3) to neutralization (15).

Assessment of ADCC requires *de novo* expression of Envelope (Env) in target cells, ideally from provirus in the context of infection, and thus cannot be done with pseudovirus. Given that utilization of replication-competent infectious molecular clones of HIV-1 strains of choice adds significant complexity to ADCC assays, the magnitude of testing described above for neutralization in TZM-bl cells would be prohibitive to implement for ADCC. Therefore, we investigated the possibility of implementing a panel of HIV-1 reference strains that reflects the global epidemic in order to facilitate testing and comparisons of ADCC responses across studies, especially for vaccine preclinical and clinical trials, which rely heavily on comparative immunogenicity data. We chose to test Envs from diverse strains (*n* = 29) encoded by infectious molecular clones with an isogenic backbone (Env-IMCs), and characterized ADCC responses against these Env-IMCs using plasma from nonmatched infected individuals (*n* = 29) in an attempt to develop standardized panels for assessment of vaccine-elicited ADCC-mediating antibody responses.

## RESULTS

### Env-IMCs broadly represent *env* diversity.

**FIG 1:**
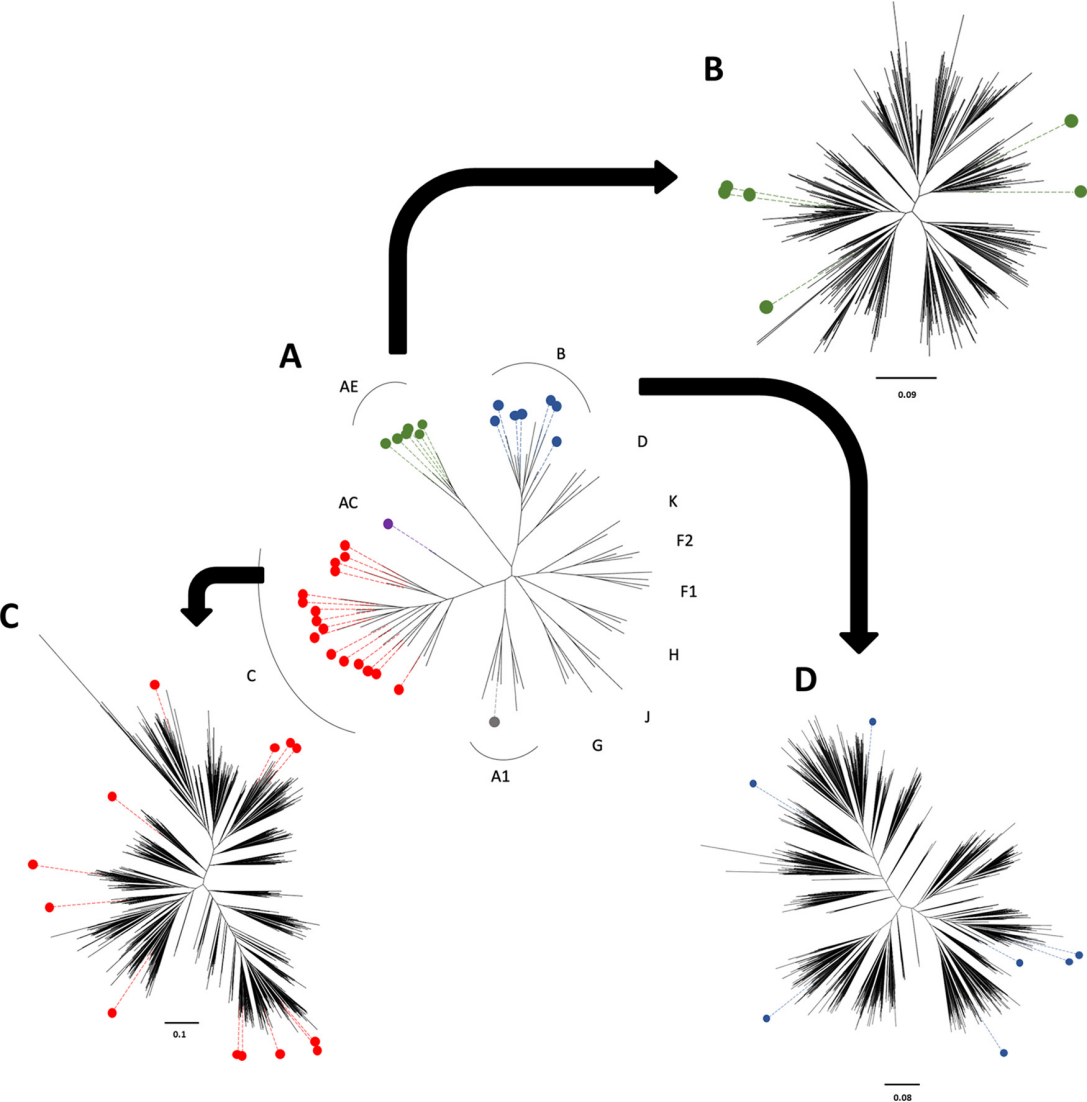
Phylogenetic trees showing the genetic diversity covered by 29 Env-infectious molecular clones (IMCs). (A) *env* ectodomains from the 29 Env-IMCs we had available were aligned with sequences representing each major subtype (four sequences per subtype), and a maximum-likelihood tree was constructed. Six CRF01 A/E sequences (green) were then aligned with 549 superfiltered subtype A/E *env* sequences (B), 16 subtype C sequences (red) were aligned with 1,315 superfiltered subtype C *env* sequences (C), and seven subtype B sequences (blue) were aligned with 1,939 superfiltered subtype B *env* sequences (D) available in the Los Alamos database, and maximum-likelihood trees were constructed to show the genetic diversity covered by the three panels.

We used 29 infectious molecular clones, each designed using an isogenic proviral backbone and encoding the ectodomain of Envs of different subtypes (Env-IMCs) for this study (Fig. 1; characteristics described in Table 1), essentially as described before (16). Phylogenetic tree analysis (Fig. 1A) illustrates that these respective *env* genes represent five different subtypes or recombinants that are responsible for the majority (∼65%) of the HIV-1 epidemic (17), namely, A1 (*n* = 1), A1C (*n* = 1), CRF01_AE (*n* = 6), B (*n* = 7), and C (*n* = 14).

**TABLE 1:** List of 29 Env-IMCs

Virus	Full Env-IMC name	Subtype	Yr isolated	Accession no. for *env* gene sequence
BaL	NL-LucR.T2A-BaL.ecto	B	1985	DQ318211
MW96.5	NL-LucR.T2A-MW96.5.ecto	C	1993	U08455
Q23.17	NL-LucR.T2A-Q23.17.ecto	A1	1994	AF004885
SF162	NL-LucR.T2A-SF162.ecto	B	1988	EU123924
TH023	NL-LucR.T2A-TH023.ecto	AE	1992	KU562843
1086.C	NL-LucR.T2A-Ce1086_B2.ecto	C	2004	FJ444395
246-F3.C10	NL-LucR.T2A-246-F3_C10_2.ecto	A1C	2000	HM215279
427299	NL-LucR.T2A-427299.c12.ecto	AE	2006	JN944655
816763	NL-LucR.T2A-816763.c2.ecto	AE	2006	JN944659
C1080.C03	NL-LucR.T2A-C1080.c03.ecto	AE	1999	JN944660
CH0505	NL-LucR.T2A-CH0505.ecto	C	2008	KC247557
CH058	NL-LucR.T2A-CH58.ecto	B	2006	EU289194
CH040	NL-LucR.T2A-CH40.ecto	B	2006	EU289193
CM235	NL-LucR.T2A-CM235-2.ecto	AE	1990	JN944662
CM244	NL-LucR.T2A-CM244.c01-ETH2220.ecto	AE	1990	KC822429
Du151	NL-LucR.T2A-Du151.2.ecto	C	1998	DQ411851
Du422	NL-LucR.T2A-Du422.1.ecto	C	1999	DQ411854
SUMA	NL-LucR.T2A-SUMA.ecto	B	1991	EU577073
TV1	NL-LucR.T2A-TV1.21.ecto	C	2002	HM215437
WITO	NL-LucR.T2A-WITO.ecto	B	2000	AY835451
YU2	NL-LucR.T2A-Yu2.ecto	B	1992	M93258
CAP210	NL-LucR.T2A-CAP210.TF.ecto	C	2005	KC894136
CAP228	NL-LucR.T2A-CAP228.2.00.18.ecto	C	2005	EF203969
CAP239	NL-LucR.T2A-CAP239.TF.ecto	C	2005	FJ443353
CAP255	NL-LucR.T2A-CAP255.2.00.16J.ecto	C	2005	EF203982
CAP256	NL-LucR.T2A-CAP256.2.00.C7.ecto	C	2005	EF203981
CAP45	NL-LucR.T2A-CAP45.2.00.G3.ecto	C	2005	DQ435682
CAP8	NL-LucR.T2A-CAP8.TF.ecto	C	2005	FJ443492
CAP88	NL-LucR.T2A-CAP88.2.00.17_5A.ecto	C	2005	EF203970

These Env-IMCs, furthermore, represented the genetic diversity among three of the major subtypes (B, C, and CRF01_AE), assessed by using subtype-specific alignments consisting of superfiltered sequences available from the Los Alamos National Laboratory database. By aligning *env* genes of the 6 CRF01_AE Env-IMCs with 549 available sequences, we found the *env* sequences of the Env-IMCs broadly represented the genetic diversity of CRF01_AE *env* genes (Fig. 1B). Similarly, the *env* genes from the 16 subtype C Env-IMCs were distributed around the tree, indicating that they represented a large degree of subtype C diversity (Fig. 1C). Last, the *env* genes of the 7 subtype B Env-IMCs we had available fell into the half of the tree generated with 1,939 subtype B sequences (Fig. 1D).

### Envelope susceptibility to ADCC and neutralization.

**FIG 2:**
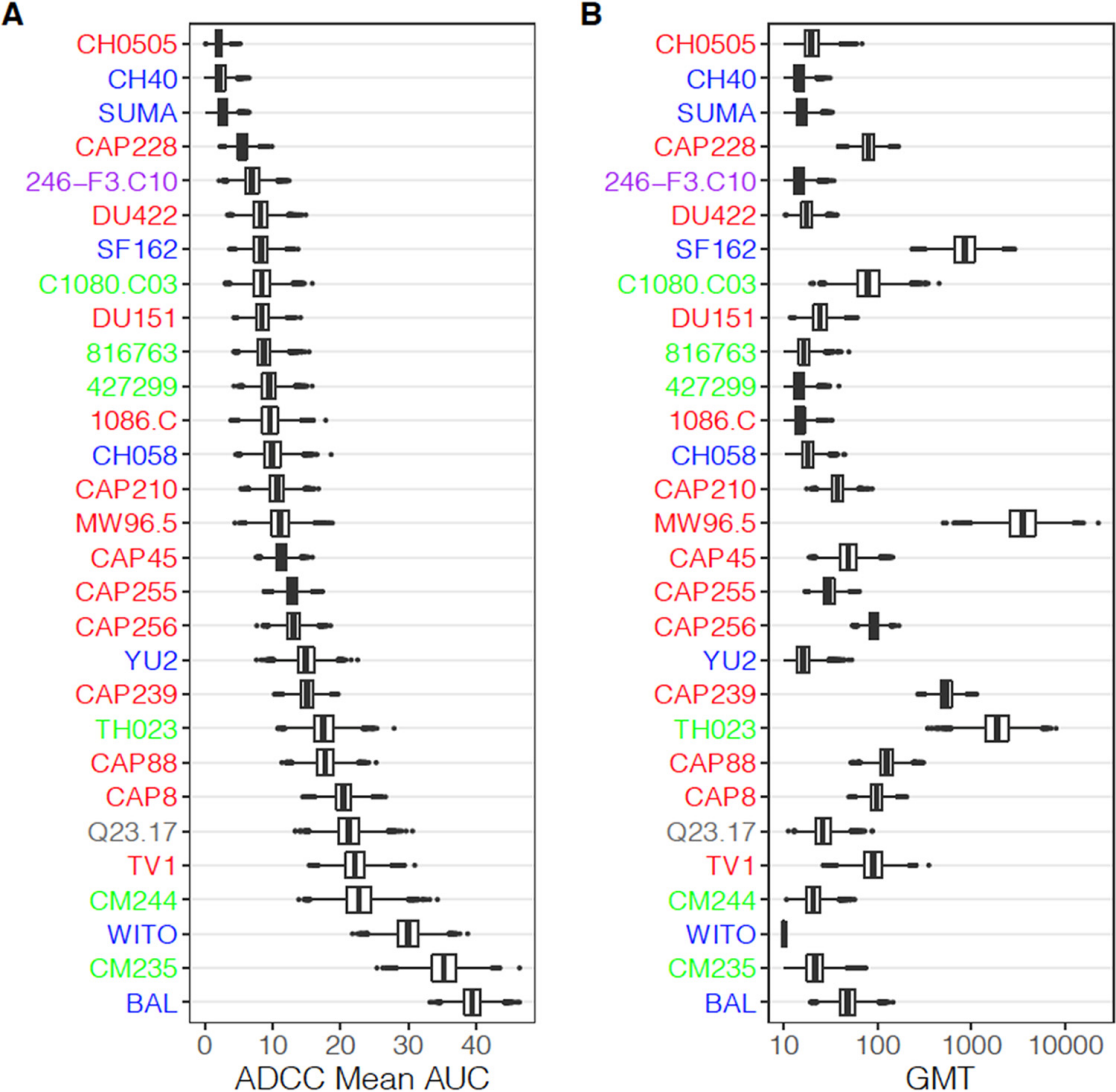
Box plots showing distribution of mean area under the curve (AUC) of ADCC and geometrical mean neutralization titer of each virus over 10,000 bootstrap samples. Viruses are sorted according to mean AUC of ADCC.

To assess the susceptibility of these viruses to ADCC, we used nonmatching plasma from 29 HIV-1-seropositive individuals infected with subtype B (*n* = 13), C (*n* = 10), or unknown subtype (*n* = 6) viruses and representing different clinical status with regard to their neutralization capacity and viral replication control, as well as plasma from three uninfected individuals as negative controls (Table 2). When we evaluated average virus susceptibility to ADCC, we observed differential recognition of viruses by ADCC-mediating plasma antibodies (Fig. 2A). For example, the subtype C Env-IMC CH0505 was extremely resistant to ADCC mediated by antibodies in plasma from the 29 individuals, with a mean ADCC area under the curve (AUC) of 3, while the subtype B Env-IMC BaL was highly susceptible to ADCC, with a mean AUC of 40.

**TABLE 2:** Plasma samples used to assess ADCC susceptibility

Neutralization profile	No. of samples of infecting strain of:		
	Subtype B	Subtype C	Unknown subtype
Weakly neutralizing	7	3	3
Controller	5	4	1
Broadly neutralizing	1	3	2

**FIG 3:**
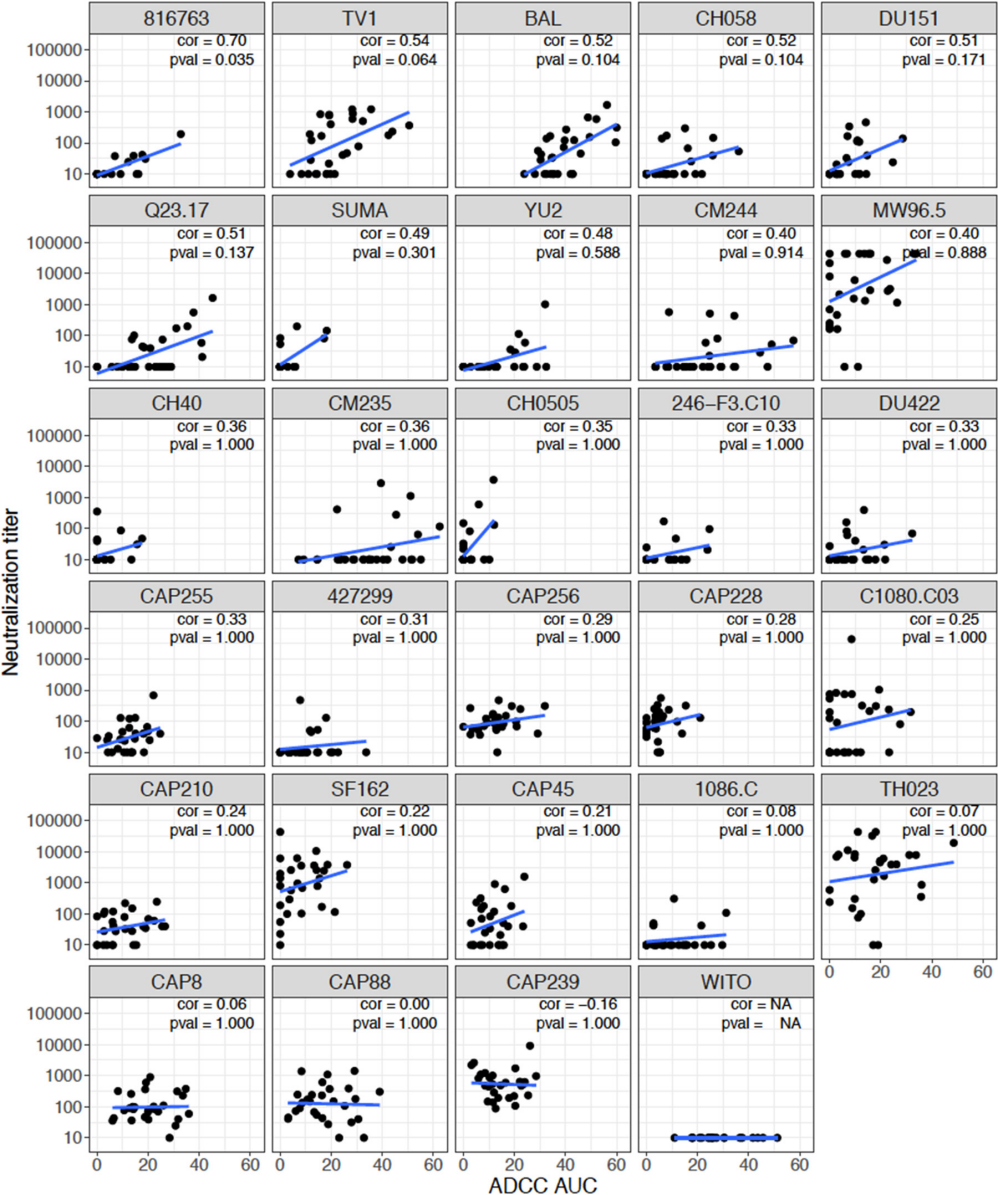
Scatterplots of neutralization titers versus ADCC (AUC) for 29 Env-IMCs. Spearman’s rank-order correlation and Bonferroni-adjusted *P* value are shown for each virus. Plots are ordered by Spearman’s rank-order correlation.

We then compared average virus susceptibility to ADCC with average virus susceptibility to neutralization using the TZM-bl neutralizing assay, shown by geometrical mean titer (GMT) for each virus in the same 29-virus panel across the set of plasma samples. Interestingly, the Env-IMCs exhibited differential susceptibility to ADCC and neutralization. The hierarchy of Env-IMC susceptibility to ADCC was not recapitulated when the same Env-IMCs were used in the TZM-bl neutralization assay and the same panel of plasma samples were used (Fig. 2). This observation was supported by a correlation analysis of the average virus susceptibility to ADCC (AUC) against average virus susceptibility to neutralization (GMTs) of the full 29-virus panel. We observed a nonsignificant positive Spearman correlation of 0.25. When looking at viruses individually, most viruses did not show a correlation between ADCC AUC and neutralization titer (Fig. 3). Responses against the CRF01_AE Env-IMC 816763 showed the highest, although moderate, Spearman correlation (*r_s_* = 0.70, *P* = 0.035), while correlations for all other Env-IMCs were less than 0.55 and tended to be positive, although none were significant after multiplicity correction.

### Effect of virus characteristics on ADCC susceptibility.

**FIG 4:**
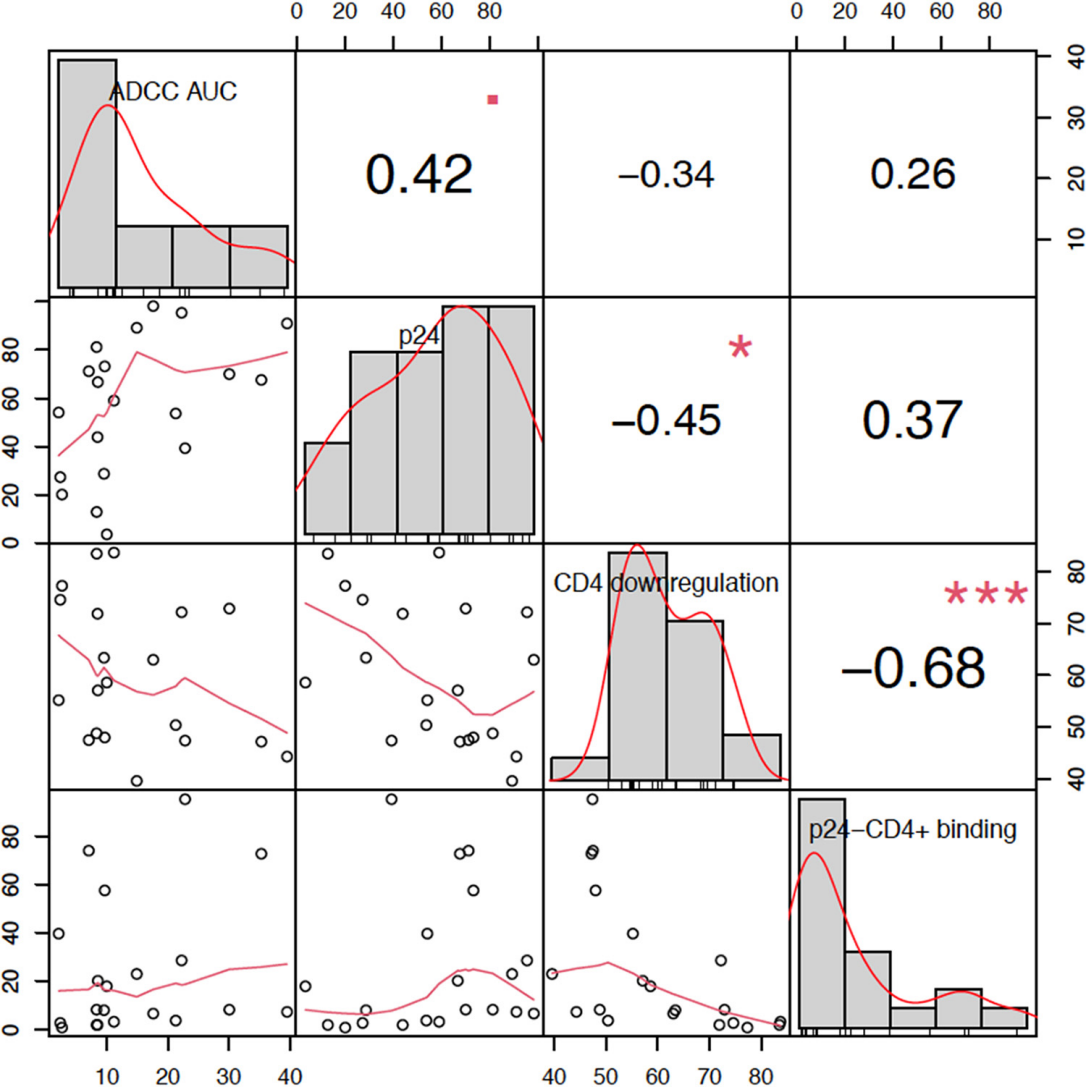
Pairwise correlation plot showing correlations between average virus susceptibility to ADCC AUC, %p24 positivity at time of ADCC assay, %CD4 downregulation, and %A32 binding to p24^−^ CD4^+^ cells for Env-IMCs in the global panel. The distribution of each variable (ADCC AUC, %p24 positivity, %CD4 downregulation, and %A32 binding to p24^−^ CD4^+^ cells) is shown on the diagonal. The bivariate scatterplots indicate the exact value for each virus with respect to the variable above in the column and the variable in the row to the left, with a fitted line displayed below the diagonal. Correlation values with the significance level represented by stars (the greater the number of stars, the higher the significance) are displayed above the diagonal.

Based upon data from 21 of the Env-IMCs, we determined the impact of several characteristics of virus-infected cells on ADCC AUC, including the degree of target cell infection (percentage of p24^+^ cells), proportion of CD4 downregulated p24^+^ cells, and level of gp120 shedding (evaluated by the percentage of A32 binding to CD4^+^ p24^−^ cells), using pairwise correlation. A modest relationship (*r_s_* = 0.42, *P* = 0.06) was observed between ADCC mean AUC and the percentage of p24^+^ cells (Fig. 4), while there was a nonsignificant moderate negative relationship between levels of CD4 downregulation and mean ADCC AUC (*r_s_* = −0.34, *P* = 0.1371). A moderate negative relationship was observed between the level of CD4 downregulation and gp120 shedding (*r_s_* = −0.68, *P* = 0.0009), indicating that as CD4 downregulation increased the level of shedding decreased, possibly due to the fact that as CD4 downregulation increases, more stable trimers are formed on the surface of the infected cell. Consequently, there are fewer unstable gp120 monomers to shed from the cell surface.

### Downselection of Env-IMCs for assessment of ADCC responses in clinical trials.

**FIG 5:**
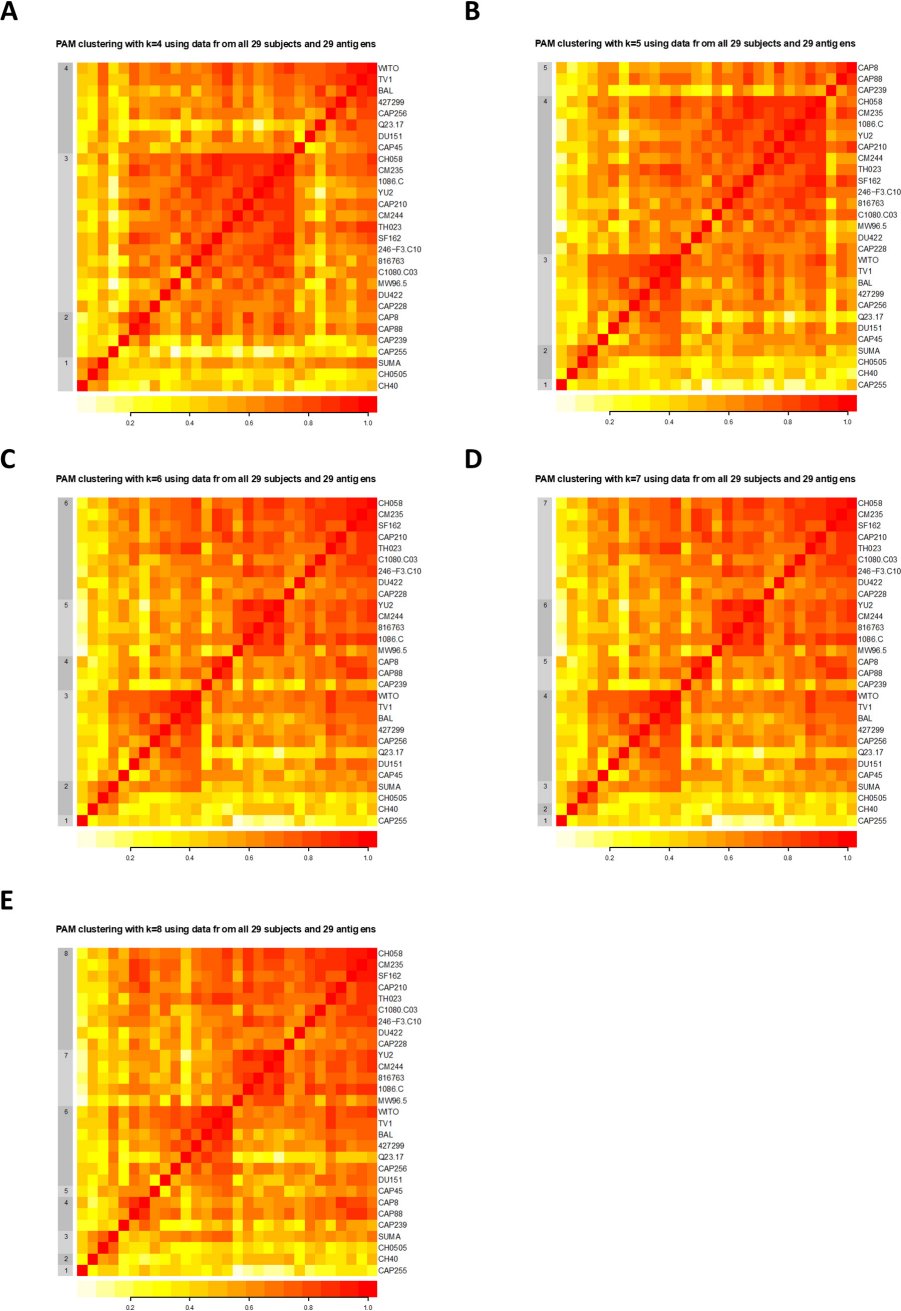
Partitioning around medoids (PAM) clustering using *k* = 4 (A), *k* = 5 (B), *k* = 6 (C), *k* = 7 (D), and *k* = 8 (E). The color scale represents the Spearman rank correlation of ADCC mediated by the 29 plasma samples against any two viruses.

**FIG 6:**
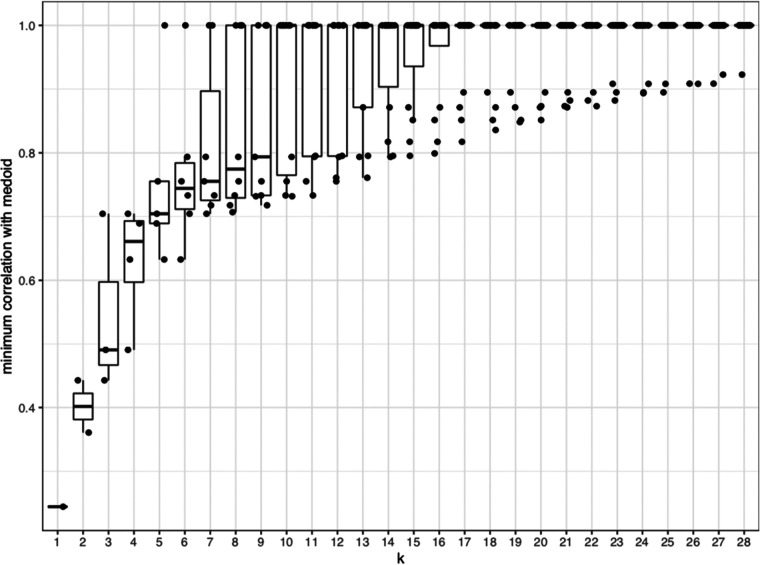
Box plots showing distribution of minimum correlation with medoid for each *k*.

After establishing the profile of susceptibility of the full Env-IMC panel to the plasma antibody (Ab) ADCC responses, we next sought to determine whether or not smaller subsets of IMCs could be used to evaluate ADCC breadth in large clinical studies. In order to achieve this goal, partitioning around medoids (PAM) clustering was used to identify groups of viruses with similar ADCC susceptibility, defined by correlation-based distances (18). PAM identifies the viruses at the center of each cluster (termed “medoids”) for a given number (*k*) of clusters, which are used to construct panels of *k* viruses representing the different clusters. Taking the need for a balanced panel into consideration, together with pragmatic factors such as the number of viruses that could realistically be tested in vaccine immunogenicity studies given the cost of the assay, we chose to focus on panels ranging between *k* = 4 and *k* = 8 viruses. We were able to identify different panels based on the number of *k*-medoids (Fig. 5). As expected, the minimum correlation of viruses within a particular cluster to the medoid increased as *k* increased (Fig. 6), such that when *k* = 4, the minimum correlation was approximately 0.5, whereas when *k* = 7, the minimum correlation was greater than 0.7. This suggested that as the number of clusters increased, each medoid better represented the ADCC susceptibility or resistance of the other viruses in the cluster. In addition, once the viruses were split into clusters of five or more medoids, we observed at least one cluster of a single virus (minimum correlation = 1), indicating that the ADCC susceptibility profile of those singleton medoids was unique enough to not cluster with any other viruses.

When dividing the 29 viruses into four clusters, the four medoids were the subtype B Env-IMCs CH058, WITO, and SUMA and the subtype C Env-IMC CAP8 (Table 3). As the number of clusters was increased to eight, these four Env-IMCs remained the same, while other Env-IMCs were added as medoids, indicating that there was a nesting of medoids as *k* increased. These data indicate that a minimum of a four Env-IMC panel could be useful for study comparison purposes, even when different numbers of viruses have been used.

**TABLE 3:** Nesting of medoids for *k* = 4 to *k* = 8 using all 29 Env-IMCs

*k* = 4	*k* = 5	*k* = 6	*k* = 7	*k* = 8
CH058	CH058	CH058	CH058	CH058
WITO	WITO	WITO	WITO	WITO
SUMA	SUMA	SUMA	SUMA	SUMA
CAP8	CAP8	CAP8	CAP8	CAP8
	CAP255	CAP255	CAP255	CAP255
		YU2	YU2	YU2
			CH40	CH40
				CAP45

### The full panel and the seven-medoid panel exhibit similar susceptibility to ADCC.

**FIG 7:**
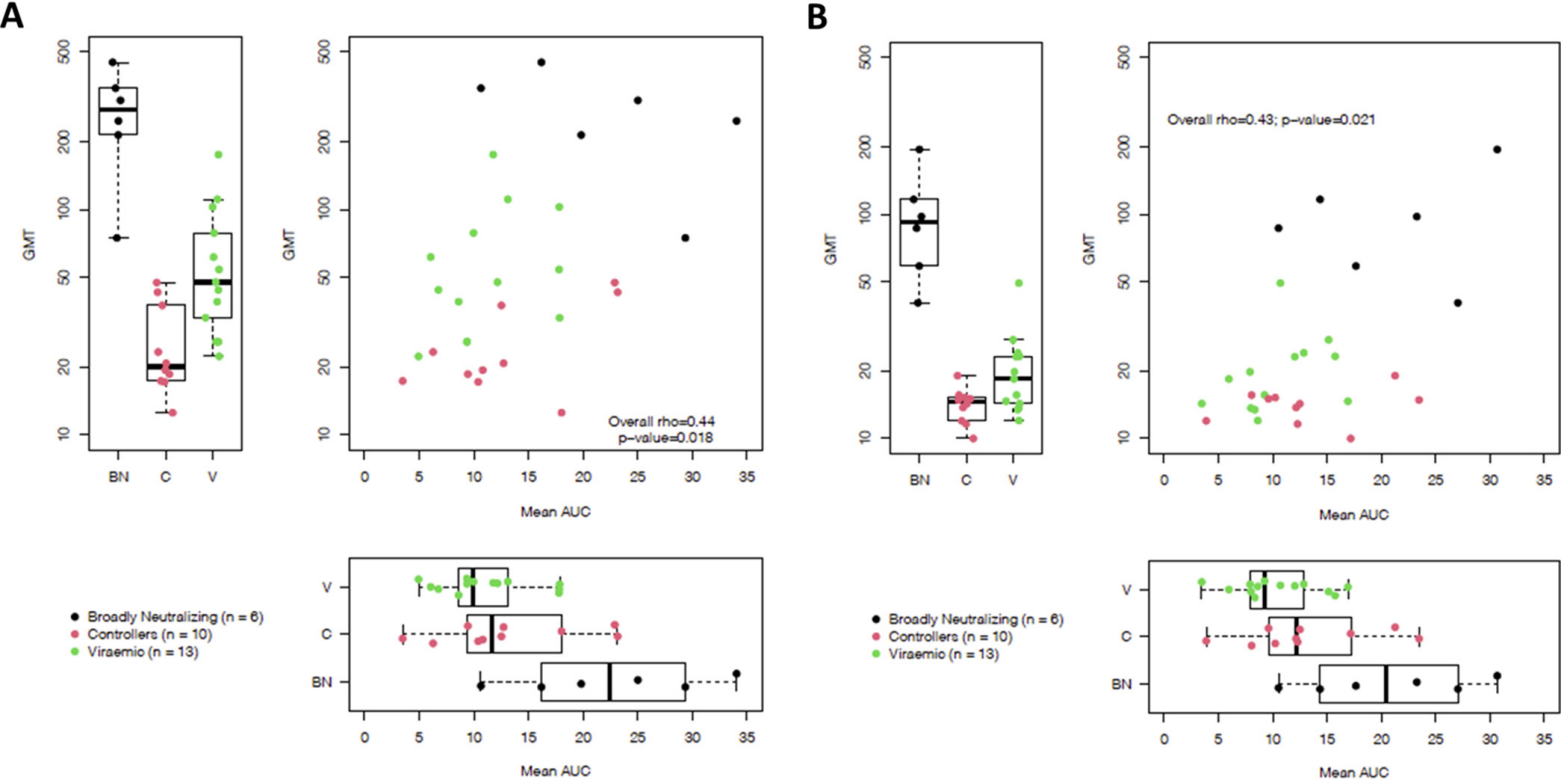
ADCC and neutralization activity of 29 plasma samples using all 29 Env-IMCs (A) and data from the 7 medoid Env-IMCs (*k* = 7) obtained using PAM (B).

To ensure that the medoids accurately represented overall patterns of ADCC activity mediated by plasma antibodies from the 29 individuals, we compared these responses against the entire 29-virus panel and the Env-IMCs representing the seven-medoid panel. We performed these analyses by simultaneously using data from both neutralization and ADCC assays to make sure that functional responses were guiding our interpretation of the panels’ comparison. When we considered the correlation of average serum neutralization potency and average serum ADCC potency against the full 29-virus panel, we observed a low positive correlation between neutralization GMT and ADCC mean AUC (*r_s_* = 0.44, *P* = 0.018) (Fig. 7A). When we investigated neutralization alone (Fig. 7A, left), we observed that broadly neutralizing samples (black) exhibited the greatest neutralization, as expected, followed by plasma from viremic individuals and then controllers. While broadly neutralizing samples also mediated the highest levels of ADCC against the 29-virus panel (Fig. 7A, bottom), ADCC mediated by antibodies in controller sample plasma was higher than that mediated by antibodies in viremic plasma samples.

These relationships remained remarkably similar when assessing the correlation of average serum-neutralizing and ADCC activity against the seven medoid viruses, where we observed a Spearman correlation of 0.43, compared to 0.44 for the full panel (*P* = 0.021 versus 0.018) (Fig. 7B). While the medoid viruses became more resistant to neutralization (Fig. 7B, left), they maintained the same susceptibility to ADCC (*P* = 0.688; Fig. 7B, bottom) suggesting an accurate representation of the full panel of 29 viruses.

## DISCUSSION

To understand how efficacious proposed HIV-1 vaccines may be, an understanding of the breadth of HIV-1 variant recognition elicited by these vaccines is crucial. In addition, it is important to be able to directly compare immune responses elicited by different vaccines when making decisions on which vaccine candidates to move forward. This requires a better understanding of the susceptibility of the Env to antibody responses to facilitate the selection of reagents dedicated to these tasks. Previously, robust statistical methods have been applied to identify a small panel of pseudoviruses that accurately represented the global epidemic for use in assessing the breadth of neutralizing antibody responses elicited by vaccine trials (19). However, there are currently no such panels of viruses for standardized assessment of vaccine-elicited ADCC-mediating antibody responses. Here, we used 29 Env-IMCs of different subtypes and sera from 29 nonmatched HIV-1-infected individuals to evaluate susceptibility of viruses produced from these IMCs to ADCC-mediating antibodies in a luciferase-based ADCC assay. Using PAM clustering, we were able to identify more economical panels of 4 to 8 Env-IMCs that accurately represented the susceptibility of the full panel of 29 Env-IMCs to ADCC responses. These panels will be essential to facilitating the standardized assessment of the breadth of vaccine-induced ADCC responses across past and future vaccine trials, in addition to the evaluation of responses to vaccine-matched reagents.

The PAM methodology used to determine appropriate panels of Env-IMCs resulted in clustering of Env-IMCs that nested for panels of size *k* = 4 to *k* = 8, such that each panel of size *k* + 1 contains all Env-IMCs in the panel of size *k*. This allows the use of 8 Env-IMCs where budget allows, while still permitting the comparison of vaccine-induced responses in other studies where fewer viruses were used. Panels derived by PAM clustering consisted entirely of subtype B and C Env-IMCs, suggesting that subtype is not a major factor in ADCC susceptibility. For example, this study suggests that little to no difference would be introduced by replacing the subtype B Env-IMC CH058 with CRF01_AE CM235, which had similar susceptibility to ADCC and correlation to other Env-IMCs in the cluster. Nevertheless, because of the relatively limited diversity of the panel, while the panel can be used for any HIV-1 vaccine, it will be most relevant for vaccines targeting regions dominated by subtype B and C and CRF01_AE strains, and it can be used to directly compare the diversity of ADCC responses across vaccines targeting the same geographic region.

The breadth of ADCC-mediating monoclonal antibodies has been reported to various degrees in numerous previous studies using both infected cells and Env-coated cells (20–24). Furthermore, we have previously characterized the breadth of ADCC responses in sera from vaccinees in HVTN100 (used as the go/no-go criteria for HVTN702 [25]) against both subtype C and CRF01_AE Env-IMCs (26). These studies suggest that using a standardized panel of viruses to characterize magnitude and breadth will improve evaluation of ADCC responses in clinical trials and provide novel insights into requirements for protection.

It is well established that neutralizing antibodies can mediate ADCC (21, 27), and a previous study found, for monoclonal antibodies, that neutralization and ADCC were generally correlated (28). While this had not previously been found using sera from infected individuals (29, 30), this may be due to the limited number of viruses used in these studies. We observed an overall positive but limited relationship between antibody neutralization and ADCC in our panel of sera, suggesting there is an overlap in neutralization and ADCC function of antibodies in polyclonal antibody responses. In addition, similarly to previous studies, we observed that plasma from broad neutralizers tended to have the highest ADCC activity (31).

However, when we ranked viruses by their susceptibility to ADCC or neutralization, we found no relationship. Viruses in this panel could be resistant to neutralization and sensitive to ADCC or vice versa, but were rarely sensitive or resistant to both responses (the highest correlation we observed between neutralization and ADCC was *r_s_* = 0.7 for Env-IMC 816763). This observation suggests there are different requirements for neutralization of virions and ADCC of virus-infected cells, which may be largely due to differences in Env epitopes targeted by neutralizing and nonneutralizing antibodies in serum. In addition, a further difference may be the presentation of Env epitopes on the membrane of virions and infected cells. Env from some viruses may spend more or less time in an “open” conformation, impacting their susceptibility to antibodies targeting CD4-induced epitopes (which are typically nonneutralizing antibodies that mediate potent ADCC) (32).

We previously reported that large portions of the HIV-1 Envelope trimer are present on the surface of target cells in a non-CD4i conformation (33), despite the *luciferase* gene included in our IMCs interfering with Nef function, resulting in incomplete downregulation of CD4 from the cell surface (34). In addition, it is important to note that the ability of Nef to downregulate CD4 differs between virus isolates (35), as does the intrinsic closed or open status of Env trimers (36, 37), making it unlikely that single-virus infection, assayed at a single time postinfection, will reflect the complexity of HIV infections *in vivo*. Furthermore, the importance of recognizing both conformations has been reported by different groups in the context of preclinical (10, 11) and clinical studies (32, 38, 39). Previous studies have identified CRF01_AE viruses as being generally more sensitive to ADCC due to the presence of a histidine residue at position 375 of the Env that fills in a phenylalanine cavity at position 43 (40). While all six CRF01_AE Env-IMCs in our panel contained histidine 375, we did not observe this association between virus subtype and ADCC susceptibility, which may be due to the characteristics of the Envs included in our panel.

Last, while subtypes B, C, and CRF01_AE account for most of the 29-Env–IMC panel, we observed no impact of subtype on susceptibility to ADCC in our analyses. In addition, we hope to construct additional Env-IMCs consisting of *env* genes from subtypes A, D, and G to more accurately represent HIV-1 genetic diversity. We intend to use the panels designed above, together with sequencing of breakthrough infections in past (including RV144 and the recently completed HVTN702) and ongoing or future vaccine trials, to fully understand the breadth and magnitude of ADCC responses being elicited by vaccines and the impact on protection.

In summary, we report here the most extensive effort to-date, using robust statistical analyses, to provide standard panels of reagents for assessing vaccine-elicited ADCC responses. We have identified panels of 4 to 8 Env strains that, expressed in the context of Env-IMCs, may be used to assess past, present, and future trials to different extents, dependent on budget, and that include *env* genes from three different subtypes (B, C, and CRF01_AE). We believe that these panels, together with reagents developed to specifically accommodate circulating viruses at the geographical sites of vaccine trials, will provide a powerful tool to harmonize generated data and detect common themes of ADCC responses elicited by various vaccines.

## MATERIALS AND METHODS

### Ethics statement.

The studies were reviewed and approved by the Duke University Medical Center Institutional Review Board, and all participants provided written informed consent. All research was performed in accordance with the Duke University School of Medicine guidelines and regulations.

### Plasma samples.

Twenty-six samples were obtained from the CHAVI 001 cohort, and three samples were obtained from the Duke controller cohort, infected by either subtype B HIV-1 (*n* = 13), subtype C HIV-1 (*n* = 10), or unknown (*n* = 6). Controllers were defined as having a viral load of <50 copies/mL, and broadly neutralizing samples were chosen from broadly neutralizing sera identified in Hraber et al. (41).

### Cell lines.

TZM-bl cells (42) were obtained from the NIH AIDS Research and Reference Reagent Program (NIH ARRRP, catalogue number 8129; contributed by John Kappes and Xiaoyun Wu). The HEK293T cell line was obtained from George Shaw (University of Pennsylvania, Philadelphia, PA). All adherent cell lines were cultured at 37°C and 5% CO_2_ in Dulbecco’s modified Eagle’s medium (DMEM) containing 10% heat-inactivated fetal calf serum (FCS; Biochrom) with 50 μg/mL gentamicin (Lonza, Basel, Switzerland) and disrupted at confluence by treatment with 0.25% trypsin in 1 mM EDTA (Lonza). CEM.NKR_CCR5_ cells were obtained through the NIH AIDS Research and Reference Reagent Program (NIH ARRRP, catalogue number 4376; contributed by Alexandra Trkola).

The CEM.NKRCCR5 cell line was used in ADCC assays to reduce variability in infections and to provide target cells that can be reproducibly used in combinations with effector cells from different donors.

### Peripheral blood mononuclear cells.

Peripheral blood mononuclear cells (PBMCs) obtained by leukapheresis from an HIV-seronegative individual (Fc gamma receptor IIIA [FcγRIIIA] 158 V/F heterozygous) and previously assessed for FcyRIIIA genotype, frequency of NK cells, and ADCC activity using control sera and monoclonal antibodies under GCLP guidelines and W oversight were used as a source of effector cells.

### Construction of HIV-1 infectious molecular clones and virus preparation.

HIV-1 infectious molecular clones (IMCs) carrying the *env* genes for different transmitted/founder viruses in an isogenic proviral backbone were constructed as previously described (16); these also expressed the *Renilla* luciferase (LucR) reporter gene under the control of the HIV-1 Tat protein. Replication-competent reporter viruses like NL-LucR.T2A-CAP239.TF.ecto and other *env*-carrying strains are collectively referred to here as Env-IMC-LucR viruses. These Env-IMC-LucR viruses were originally designed to preserve all nine viral open reading frames linking Nef expression to LucR via a ribosome-skipping T2A peptide, but we later found that Nef expression of these viruses was reduced in primary CD4 T cells (43). Replication-competent viruses were obtained by transfecting the Env-IMC-LucR plasmid in HEK293T cells using PolyFect transfection reagent (Qiagen). Virus-containing supernatants were harvested 48 h following transfection, clarified by 0.45-μm filtration, and adjusted to 10% FCS (Biochrom). The 50% tissue culture infectious dose (TCID_50_) for each IMC preparation was determined by infection of TZM-bl cells as previously described (44, 45).

Viruses were then used to infect CEM.NKR_CCR5_ cells, which were characterized for suitability in a *Renilla* luciferase ADCC assay using criteria previously determined in assay qualification for use in clinical trial immunogenicity assessments. These criteria included ensuring >5% p24^+^ (infected) cells after infection and assessing the level of CD4 downregulation induced by infection of cells (Fig. 4).

### Infection of the CEM.NKR_CCR5_ cell line with HIV-1 Env-IMC-LucR.

A total of 1 × 10^6^ CEM.NKR_CCR5_ cells (NIH AIDS Reagent Program, Division of AIDS, NIAID, NIH; from Alexandra Trkola) were infected with virus inoculum at a titer determined to achieve at least 5% infection in the presence of DEAE-dextran (7.5 μg/mL). The cells were subsequently resuspended at 0.3 × 10^6^/mL and cultured for 3 days in complete medium containing 7.5 μg/mL DEAE-dextran. On the day of assay, the infection was monitored by measuring the frequency of cells expressing intracellular p24. Assays performed using the Env-IMC-LucR-infected target cells were considered reliable if the percentage of viable p24^+^ target cells on the day of assay was ≥5%. Following 72 h of infection, cells were washed in phosphate-buffered saline (PBS), dispensed in 96-well V-bottomed plates at 1 × 10^5^ viable cells per well, and stained with a vital dye (Live/Dead Fixable Aqua dead cell stain; Invitrogen) to exclude nonviable cells from subsequent analyses. The cells were then washed with washing buffer (WB; PBS plus 1% FBS) and incubated with an anti-CD4 antibody (clone OKT4-PerCP-cyanine 5.5; BioLegend) at 1:40 for 20 min at room temperature. Cells were then washed with WB again, incubated with Cytofix/Cytoperm (BD Bioscience) for 20 min at 4°C, and washed with 1% Cytoperm washing buffer (BD Biosciences). After the final wash, the anti-p24 antibody (clone KC57-RD1; Beckman Coulter) was added to a final dilution of 1:400, and the plates were incubated for 30 min at 4°C. The plates were washed twice with Cytoperm washing buffer, and the cells were resuspended in 1% formaldehyde-PBS. The samples were acquired within 24 h using a BD Fortessa flow cytometer. A minimum of 10,000 total singlet events was acquired for each analysis. Gates were set to include singlet and live events. The appropriate compensation beads were used to compensate the spillover signal for the three fluorophores. Data analysis was performed using FlowJo 10.2 software (BD Biosciences). Uninfected CEM.NKR_CCR5_ and chronically infected A1953 cell lines were used to titer the vital dye and anti-p24 antibody and appropriately determine the position of the CD4^+^/CD4^−^ gate and were used as negative and positive controls, respectively, for the described staining procedure.

### Neutralization assay.

Neutralization was measured, as previously described (44), by a reduction in luciferase gene expression after single round infection of TZM-bl cells with Env pseudoviruses or infectious viruses. Titers were calculated as the reciprocal plasma dilution that resulted in a 50% inhibitory dose (ID_50_) of the relative light units (RLU).

### *Renilla* luciferase-based ADCC assay.

The LucR-based ADCC assay was conducted as described by Pollara et al. (46). The day prior to the ADCC assay, cryopreserved PBMCs to be used as effectors in the assay were thawed in R10, counted and assessed for viability, and resuspended in R10 overnight. On the day of the assay, infected CEM.NKR_CCR5_ cells were counted, assessed for viability (viability ≥ 80%), and the concentration was adjusted to 2 × 10^5^ viable cells/mL (5 × 10^3^ cells/well). PBMCs were then counted, assessed for viability, pelleted, and resuspended in the infected CEM.NKR_CCR5_ cells at a concentration of 6 × 10^6^ PBMCs/mL (1.5 × 10^5^ PBMCs/well) (effector:target cell ratio of 30:1). Heat-inactivated autologous plasma was serially diluted. The effector/target cell mix and antibody dilutions were plated in opaque 96-well half-area plates, centrifuged at 300 × *g* for 1 min after 30 min incubation at room temperature, and then incubated for 5.5 h at 37°C and 5.5% CO_2_ to allow ADCC-mediated cell lysis to proceed. After 5.5 h, ViviRen substrate (Promega) was diluted 1:500 in R10 and added 1:1 to the assay wells. The substrate generates luminescence only in live, infected cells, not in dead or lysed cells. The final readout was the luminescence intensity generated by the presence of residual intact target cells that had not been lysed by the effector population in the presence of ADCC-mediating antibodies. The percent specific killing was calculated using the following formula:

$$\%\text{ specific killing}=\frac{\text{RLU of target well}+\text{RLU of effector well}-\text{RLU of test well}}{\text{RLU of target well}+\text{RLU of effector well}}\times 100$$

In the analysis, the RLU of the target and effector wells represent lysis by NK cells in the absence of any source of antibody. Plasma from three seronegative donors were used as a negative control, and plasma from a chronically infected individual was used as a positive control. Titers were calculated as the reciprocal of the highest plasma dilution that resulted in a percentage of specific killing above the background signal.

### Statistical analyses.

**(i) ADCC AUC.** The specific killing activities in the ADCC assay were summarized for each subject and antigen by computing the area under the dilution curve (AUC) as the mean of the specific killing activities across the dilution series. Specific killing activities less than 15 were assigned a value of 0 prior to AUC calculation.

### (ii) NAb ID_50_ titers.

ID_50_ titers from the neutralization assay that were lower than the limit of detection of 20 were assigned a value of 10.

### (iii) Bootstrapped confidence intervals.

Confidence intervals (95%) for the antigen-specific mean ADCC AUCs and geometrical mean neutralizing antibody (NAb) ID_50_ titers (GMTs) across the 29 seropositive plasma samples were computed from 10,000 bootstrapped samples generated with replacement.

### (iv) Correlation analysis.

Spearman’s rank-order correlations (*r_s_*) were computed between ADCC AUCs and NAb GMTs for each antigen using only pairwise complete observations. The significance of correlation coefficients was adjusted using Bonferroni correction. Spearman’s rank-order correlations were also computed between mean AUCs, percent p24 positivity at time of ADCC assay, percent CD4 downregulation, and percent A32 binding to p24^−^ CD4^+^ cells for antigens in the global panel.

### (v) Clustering analysis.

Using ADCC AUC data from 29 plasma samples, a matrix of Spearman rank correlations was generated between all pairs of 29 Env-IMCs, which was subsequently used to create a distance matrix using 1 minus the correlation. Based on the distance matrix, *k* clusters were formed in which *k* ranged from 1 to 28, using the method of partitioning around medoids (PAM) from the *cluster* package in R (M. Maechler, P. Rousseeuw, A. Struyf, M. Hubert, and K. Hornik, “Cluster analysis basics and extensions.” R package version 2.0.5). For each *k*, the proposed panel of size *k* was defined as the *k* medoids identified by the PAM algorithm. The distance matrix was also used to create heatmaps to visually depict the clusters and the medoids identified by PAM. Within each *k* cluster, the minimum correlation between antigens and between antigens and medoid were used to identify the optimal *k* medoids, or the minimal subset of antigens that could be used to represent the complete panel.

### Data availability.

Envelope sequences of all Env-IMCs are accessible through GenBank; accession numbers are provided in Table 1. Other data and reagents are available upon request.
